# Co and Co_3_O_4_ in the Hydrolysis of Boron-Containing Hydrides: H_2_O Activation on the Metal and Oxide Active Centers

**DOI:** 10.3390/ma17081794

**Published:** 2024-04-13

**Authors:** Vladislav R. Butenko, Oksana V. Komova, Valentina I. Simagina, Inna L. Lipatnikova, Anna M. Ozerova, Natalya A. Danilova, Vladimir A. Rogov, Galina V. Odegova, Olga A. Bulavchenko, Yuriy A. Chesalov, Olga V. Netskina

**Affiliations:** 1 Boreskov Institute of Catalysis SB RAS, 5 Akademika Lavrentieva Ave., Novosibirsk 630090, Russia; vladislav.but@mail.ru (V.R.B.); simagina@catalysis.ru (V.I.S.); lil@catalysis.ru (I.L.L.); ozerova@catalysis.ru (A.M.O.); n.danilova1@g.nsu.ru (N.A.D.); rogov@catalysis.ru (V.A.R.); odegova@catalysis.ru (G.V.O.); obulavchenko@catalysis.ru (O.A.B.); chesalov@catalysis.ru (Y.A.C.); netskina@catalysis.ru (O.V.N.); 2 Department of Natural Sciences, Novosibirsk State University, 1 Pirogova Str., Novosibirsk 630090, Russia

**Keywords:** cobalt, cobalt oxide, sodium borohydride, ammonia borane, ethylenediamine bisborane, hydrolysis, activation, hydrogen production, rate-determining step, DFT

## Abstract

This work focuses on the comparison of H_2_ evolution in the hydrolysis of boron-containing hydrides (NaBH_4_, NH_3_BH_3_, and (CH_2_NH_2_BH_3_)_2_) over the Co metal catalyst and the Co_3_O_4_-based catalysts. The Co_3_O_4_ catalysts were activated in the reaction medium, and a small amount of CuO was added to activate Co_3_O_4_ under the action of weaker reducers (NH_3_BH_3_, (CH_2_NH_2_BH_3_)_2_). The high activity of Co_3_O_4_ has been previously associated with its reduced states (nanosized CoB_n_). The performed DFT modeling shows that activating water on the metal-like surface requires overcoming a higher energy barrier compared to hydride activation. The novelty of this study lies in its focus on understanding the impact of the remaining cobalt oxide phase. The XRD, TPR H_2_, TEM, Raman, and ATR FTIR confirm the formation of oxygen vacancies in the Co_3_O_4_ structure in the reaction medium, which increases the amount of adsorbed water. The kinetic isotopic effect measurements in D_2_O, as well as DFT modeling, reveal differences in water activation between Co and Co_3_O_4_-based catalysts. It can be assumed that the oxide phase serves not only as a precursor and support for the reduced nanosized cobalt active component but also as a key catalyst component that improves water activation.

## 1. Introduction

Hydrogen is a perfect candidate for future fuel use due to its high energy density, oxidation product (H_2_O), environmental friendliness, and the possibility of H_2_ recovery from water using renewable energy sources [1,2]. A significant challenge with hydrogen energy lies in its storage and transportation. Compressed gaseous and liquid H_2_ do not meet the requirements for safety and compactness. This leads to the development of compact hydrogen storage and generation systems based on adsorbed or chemically bonded states [3,4]. For example, solid hydride compounds are actively studied. Among them, sodium borohydride (NaBH_4_, SBH) [5], ammonia borane (NH_3_BH_3_, AB) [6,7], and ethylenediamine bisborane ((CH_2_NH_2_BH_3_)_2_, EDBB) [8,9] are characterized by high values of hydrogen density (10.8, 19.6, and 16.3 wt%, respectively). Resistance to air moisture, which increases in the row SBH < AB < EDBB, makes them promising for storing and generating hydrogen in fuel cell-based mobile energy devices.

Catalytic hydrolysis of these hydrides is widely studied [10] because it produces H_2_ at ambient temperatures (1)–(3) [11,12]:

NaBH_4_ + 4H_2_O → NaB(OH)_4_ + 4H_2_ Δ_r_H^0^_298_ = −217 kJ/mol W_m_ = 10.2 wt%, (1)

NH_3_BH_3_ + 3H_2_O → NH_3_ + B(OH)_3_ + 3H_2_ Δ_r_H^0^_298_ = −156 kJ/mol W_m_ = 8.5 wt%, (2)

and (CH_2_NH_2_BH_3_)_2_ + 6H_2_O → (CH_2_NH_2_)_2_ + 2B(OH)_3_ + 6H_2_ W_m_ = 7.1 wt% (3)

where W_m_ is the gravimetric hydrogen capacity of the hydrogen-generating system calculated from the stoichiometry of the reactions (1)–(3).

The catalyst is an important control tool for H_2_ generation, and the addition of water to the solid-phase composition of the hydride and catalyst can be considered the easiest way to generate H_2_ on demand [13,14]. The study of the catalytic hydrolysis of AB and EDBB is significant also because it explores the potential for a two-stage hydrothermolysis process. This process operates under a limited water supply, where the heat generated from the exothermic hydride hydrolysis warms the reaction layer. This, in turn, triggers a low-temperature solid-phase hydride dehydrogenation that occurs in the absence of water [15,16].

In most cases, the literature presents data on the high Co_3_O_4_ activity in SBH hydrolysis [17,18,19,20,21,22]. In the reaction medium of such a strong reducing agent, in situ activation of Co_3_O_4_ occurs through its partial reduction, forming catalytically active centers. Many techniques have been applied to study the in situ-produced catalytically active phase during the reduction in cobalt compounds, including Co_3_O_4_ [23]. As a result, amorphous nanoparticles of cobalt borides (Co_x_B) [17], metal clusters coated with an amorphous layer containing Co_x_B [24], and metal cobalt trimers stabilized in a borohydride matrix [25] have been proposed as catalytically active phases. Until recently, Co_3_O_4_ activity in SBH and AB hydrolysis has been associated only with reduced nanosized cobalt forms (Co^0^, Co_x_B then be referred to as CoB_n_) [23]. Based on traditional knowledge of electroless plating techniques, NaBH_4_ often exhibits pronounced boron deposition. The application of boranes enables the production of metal depositions devoid of even trace amounts of boron. The impossibility of forming active centers under the action of EDBB seems to explain the lack of reports of Co_3_O_4_ activity in the hydrolysis of this hydride.

In the last two to three years, a current approach to designing boron-containing hydride hydrolysis catalysts involves producing bifunctional catalysts [26,27], whose active centers should activate both hydride (4) and water (5).

R-H + 2* = R* + H*, where R = BH_3_, NH_3_BH_2_, BH_3_NH_2_CH_2_CH_2_NH_2_BH_2_ (4)

H_2_O + 2* = OH* + H* (5)

Correlations between the experimentally measured catalyst activity and the DFT-calculated energy parameters of the reagents activation ((4) and (5)) on the catalyst surface suggest that the breakage of the O-H bond of the water molecule (5) on the metal active center can be considered as a rate-determining step (RDS). DFT results confirm that activating water on a metal surface requires overcoming a higher energy barrier compared to the hydride activation stage [28,29,30]. Additionally, the published results on the kinetic isotopic effect measurement (KIE, k_H_/k_D_) for the hydrolysis of AB and SBH in the case of replacing H_2_O with D_2_O (Table S1 in the Supplementary Materials) also show that the breakage of the O-H bond of the water molecule (5) on the metal active center can be considered as an RDS.

From this point of view, it is interesting to discuss about the catalytic properties of Co_3_O_4_ again. The published works make it possible to state that without the formation of a sufficient content of the nanosized metal-like phase (CoB_n_), hydrolysis of hydride is not carried out, which is due to the low rate of B-H bond activation on the catalyst surface. However, during intense hydrogen evolution, the degree of Co_3_O_4_ reduction in the reaction medium is uncertain. The characteristics of the unreduced residual cobalt oxide phase and its influence on the kinetic regularities are also under discussion.

It is well known that Co_3_O_4_ treatment with sodium borohydride in aqueous solution is widely used to form anionic vacancies (V_o_) in the oxide structure, which has improved the characteristics of catalysts and materials for various applications [31,32,33,34,35]. The results obtained in different research groups are consistent. Though the main crystalline phase remains Co_3_O_4_, there is an increase in the Co^2+^/Co^3+^ ratio on the surface of the treated sample, with a simultaneous increase in the content of anionic vacancies. In most studies, the relative content of anionic vacancies is judged by the results of O 1s XPS spectra deconvolution, where the ratio of the content of lattice oxygen species and surface adsorbed oxygen is estimated.

The first report on the bifunctional properties of activated Co_3_O_4_ was published in 2020 [36]. In this study, the authors, when explaining the high catalytic activity of Co_3_O_4_/CN (CN = carbon nitride) in AB hydrolysis, underlined the importance of Co^0^ in the activation of AB (4) and Co_3_O_4_ in the process of water activation (5). They have used DFT modeling to calculate the adsorption energy (E_ads_) and activation energy (E_a_) for the dissociative adsorption of water (5) on CN-supported clusters of Co^0^, Co_3_O_4_, and Co_3_O_4_ with introduced anion vacancies in its structure (Co_3_O_4_-V_O_). As expected, water activation on the oxides was shown to be more effective than on the metals. But, the calculations in this paper demonstrate that the formation of anionic vacancies in Co_3_O_4_-V_O_ did not decrease the value of E_ads_ and E_a_ compared to Co_3_O_4_. However, the opposite results on the influence of oxygen vacancies in the Co_3_O_4_ structure on E_ads_ have been demonstrated in [37,38].

Therefore, this work will focus on the comparison of the kinetics of H_2_ evolution in the hydrolysis of boron-containing hydrides of different natures and reducing abilities (NaBH_4_, NH_3_BH_3_, and (CH_2_NH_2_BH_3_)_2_) over the ex situ-formed cobalt metal catalyst and the Co_3_O_4_-based catalysts whose activation proceeded in the reaction medium (in situ). In the beginning, DFT calculations for metal clusters of Co, Ni, and Cu will be used to emphasize the importance of the water activation stage in the processes under study and get the answer to why Co^0^ is considered the most active metal in these processes. The kinetic regularities of the Co^0^ catalyst in the hydrolysis of the hydrides with KIE measurement at replacing H_2_O with D_2_O will then be obtained to confirm the DFT results. Subsequently, the results of the activation of Co_3_O_4_ in different hydride environments will be presented, and the effect of the reducing ability of hydrides on the hydrogen generation rate will be discussed. To increase the reduction degree of Co_3_O_4_ in the reaction medium of AB and EDBB, the studied cobalt oxide will be modified with CuO. It has been established that the contribution of Cu to the overall process can be ignored. To characterize the oxide phase in activated Co_3_O_4_, a Co_3_O_4_ sample will be removed from the SBH reaction medium and studied using several methods (TEM, TPR H_2_, XRD, Raman, and ATR FTIR). Within the one DFT model, the energy parameters (E_a_, E_ads_) of the dissociative adsorption of water on the clusters of Co_3_O_4_ and Co_3_O_4_ with two types of anionic vacancies will be compared. These results, supported by the KIE measurements in the hydrolysis reaction tests, will be compared with the data for the Co^0^ catalyst. This will reveal the differences in the water activation between a cobalt metal catalyst and a catalyst containing a cobalt oxide phase.

## 2. Materials and Methods

### 2.1. Catalytic Materials under Study

A commercial sample of Co_3_O_4_ (clean for analysis, GOST 4467-79, SoyuzKhimProm, Novosibirsk, Russia) was used. To modify Co_3_O_4_ with CuO (10 wt%), 4.0053 g of Co_3_O_4_ and 0.6123 g of CuCO_3_·Cu(OH)_2_ (clean, GOST 8927-79, Reachem, Moscow, Russia) were ground in a hand mortar and the mixture was calcined at 300 °C for 4 h.

The metal cobalt catalyst was prepared by the galvanic replacement method, as previously reported [39]. Aluminum powder (ASD-0 grade, TU 1791-007-49421776-2011, Sual-PM, Shelekhov, Russia), with an average particle size of 80 ± 18 μm, was used as a template. Initially, 0.5 g of Al powder was degreased in acetone and etched in a 1 M HCl solution (5 mL) to eliminate the surface oxide layer. Next, 15 mL of a 0.23 M solution of cobalt (III) acetylacetonate (pure, TU 6-09-09-520-73, Reakhim, Moscow, Russia) in ethanol was added to the Al suspension. The reaction was carried out in an ultrasonic bath (Sapfir, Moscow, Russia) at 60 °C and 100 W for 2 h. The resulting sample was separated from the reaction medium with a magnet, washed with distilled water, and treated with a 2.5 M NaOH solution for 2 h to remove the residue of Al. Afterward, it was washed with distilled water until a neutral pH was achieved, rinsed with acetone, and then evacuated for 2 h at room temperature. Based on elemental analysis and XRD data, the resulting cobalt catalyst contains 98.1 wt% of Co and 0.06 wt% of Al and consists of one crystalline phase of metallic cobalt (hcp) with a CSR of 12 nm.

The scanning electron microscopy images of Co_3_O_4_, 10% CuO-90% Co_3_O_4_, and Co^0^ are presented in Figure S1 in the Supplementary Materials.

### 2.2. Experimental Methods of Investigations

X-ray diffraction analysis (XRD) was performed on a high-resolution STOE Stadi MP diffractometer in a transmission geometry (STOE & Cie GmbH, Darmstadt, Germany) in the range of angles 2–50° with a step 2θ = 0.015° using a Mythen2 1K (Dectris, Baden-Daettwil, Switzeland). MoK_α1_ radiation (λ = 0.709 Å) was used. A phase analysis was performed by the Rietveld method. The average coherent scattering regions (CSR) were determined using the Scherrer formula from the following reflections: 111 for CoO and 311 for Co_3_O_4_. The phases were identified using the following data: CoO [PDF 42-1300] and Co_3_O_4_ [PDF 42-1467].

Temperature-programmed reduction with hydrogen (TPR H_2_) was carried out in a custom-built unit equipped with a flow quartz reactor and a thermal conductivity detector. Before the experiment, the cobalt oxide sample (7 mg) was mixed with quartz sand and purged with argon for 2 h at room temperature. A gas mixture of 10 vol.% H_2_ in Ar was supplied at a rate of 40 mL/min. The rate of heating from room temperature to 900 °C was 10 K/min.

The Raman spectrometer T64000 (Horiba Jobin Yvon, Edison, NJ, USA) with a micro-Raman setup was used to measure the Raman spectra. All experimental spectra were collected in the backscattering geometry using the 514.5 nm line of an Ar^+^ laser. The spectral resolution was not worse than 1.5 cm^−1^. The detector was a silicon-based CCD matrix cooled with liquid nitrogen. The power of the laser beam reaching the sample was 2 mW. The band at 520.5 cm^−1^ of Si single crystal was used to calibrate the spectrometer.

High-resolution transmission electron microscopy (HR TEM) studies were carried out using a JEM-2010 instrument (JEOL Ltd., Akishima, Japan) with a lattice resolution of 1.4 Å and an accelerating voltage of 200 kV. Before the experiment, the samples were fixed on “holey” carbon films supported by copper grids.

Attenuated total reflection infrared spectroscopy (ATR FTIR) was performed on an Agilent Cary 630 (Agilent Technologies, Santa Clara, CA, USA) spectrometer in the range of 540–4000 cm^−1^.

The scanning electron microscopy (SEM) images were obtained with a JEOL JSM-6460 LV (Jeol, Akishima, Japan) instrument.

### 2.3. Catalytic Hydrolysis of Boron-Containing Hydrides

For catalytic experiments, commercial NaBH_4_ (98%, CAS 16940-66-2, Chemical Line, Sankt-Petersburg, Russia) was used. Synthesis of NH_3_BH_3_ with 97% purity and (CH_2_NH_2_BH_3_)_2_ with 96% purity was performed according to well-known [8,40] (see Supplementary Materials). The phase composition of the synthesized hydrides was confirmed by XRD and ATR FTIR methods. The purity of hydride samples was estimated in a catalytic hydrolysis experiment in the presence of a preliminary reduced cobalt catalyst, taking into account the stoichiometry of reactions ((2) and (3)).

The SBH, AB, and EDBB hydrolysis was carried out at 40 °C under 750 rpm stirring. First, 10 mL of distilled water was added into a glass reactor (V = 52.5 mL) and preheated to the required temperature; then, the hydride and the catalyst powder were added in succession. The reactor was sealed, and the evolving hydrogen was passed through a condenser to the gas burette (100 mL) equipped with digital pressure Sendo sensor SS312 (Sendo sensor, Shenzhen, China). The tested amount of hydride sample was 35 mg (0.925 mmol) for SBH, 38 mg (1.23 mmol) for AB, and 54 mg (0.615 mmol) for EDBB. In all cases the initial concentration of B-H bond in the reaction medium was 3.7 mmol in 10 mL of solution. In all experiments, the amount of catalyst was 11.7 mg. The obtained values of hydrogen volume were reduced to the N.T.P.

### 2.4. Density Functional Theory Calculations

As the surfaces under investigation, the 111 facets for metals and CuO and the 311 facets for Co_3_O_4_ were selected. The adsorption of H_2_O on the surfaces of metals and oxides was studied using density functional theory (DFT) calculations, conducted within the standard Kohn–Sham formalism employing a plane-wave basis set with a kinetic energy cutoff of 350 eV. These calculations were carried out using the Vienna ab initio simulation package (VASP). The Perdew–Burke–Ernzerhof (PBE) functional of the generalized gradient approximation (GGA) was chosen for the exchange–correlation potential calculations, disregarding van der Waals interactions. The influence of atomic nuclei on the electron density was accounted for by the projector augmented wave (PAW) method. For integration in reciprocal space, a Monkhorst–Pack grid of (2 × 2 × 1 k-points) was utilized. The conjugate gradient algorithm, with an energy convergence criterion of 1 × 10^−5^ eV, was employed to ensure the convergence of atomic positions, ensuring that the forces were less than 2 × 10^−2^ eV/Å.

The adsorption energy (E_ads_) between the adsorbate molecule and the adsorbent surface was calculated as follows:

E_ads_ = [E_AB_ − (E_A_ + E_B_)], (6)

where E_AB_ represents the total energy of the surface with the adsorbed molecule A, E_A_ is the total energy of the adsorbate molecule, and E_B_ is the total energy of the adsorbent surface.

The energy (ΔE) of dissociative adsorption was calculated as follows:

ΔE = E_FS_ − E_IS_, (7)

where E_FS_ and E_IS_ are the final and initial energies, respectively.

The calculation of activation energies was performed using the nudged elastic band method with a climbing image (NEB-CI), where preoptimized structures were employed as initial and final points for the reaction pathway calculations ((4) and (5)). These calculations were continued until the magnitude of the forces orthogonal to the reaction coordinate was reduced to less than 0.05 eV/Å. The activation energy was calculated using the formula:

E_a_ = E_TS_ − E_IS_, (8)

where E_TS_ is the energy of the transition state.

Bader analysis was conducted using the code developed by Professor G. Henkelman’s group [41].

## 3. Results

### 3.1. The Characteristics of Reactions When Co^0^ Is Used as a Catalyst

#### 3.1.1. DFT Modeling of H_2_O and NH_3_BH_3_ Activation on Metallic Surfaces of Co, Ni, and Cu

To reveal the RDS of hydrolysis of boron-containing hydrides, the dissociative adsorption processes of AB (4) and water (5) on the 111 surfaces of metal clusters of Co, Ni, and Cu were studied (Figure S2). Ni and Cu are taken as comparison metals. Adsorption has been found to be characterized by insignificant changes in the geometry of both the metal surface and the adsorbate molecules. At the same time, the increase in the values of E_ads_ of H_2_O is observed in a row: Co < Ni < Cu (Table 1). This indicates a decrease in its bonding strength when moving from Co to Cu. This row is also observed when comparing E_ads_ for AB. But, in the case of AB, the effect of metal nature on E_ads_ values is more pronounced, which is reflected in more noticeable changes in the geometry of the adsorbed hydride molecule (Table 1).

**Figure 1:**
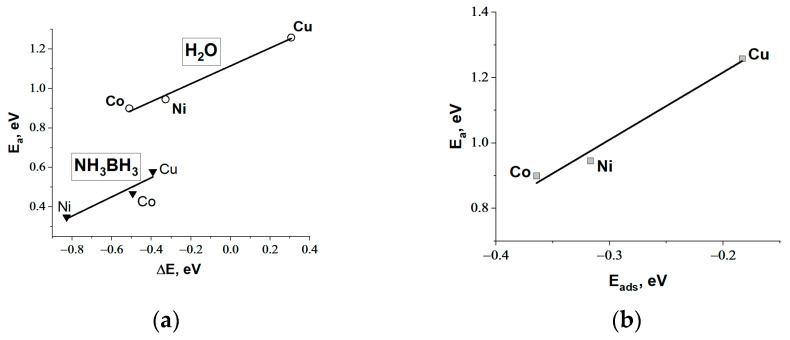
(**a**) The correlation between ΔE and E_a_ (the Brønsted–Evans–Polanyi principle) for dissociative adsorption of NH_3_BH_3_ (4) and H_2_O (5), and (**b**) the correlation between E_ads_ and E_a_ (the Sabatier principle) for dissociative adsorption of H_2_O (5) on different metal surfaces.

Using preoptimized initial and final states (Figure S2), the reaction profiles for the B-H bond cleavage in the AB molecule (4) and the O-H bond cleavage in the water molecule (5) were constructed through interpolation methods. Local maxima along the reaction coordinate were identified using the NEB-CI method, and the activation energy (E_a_) for each metal was determined. The obtained results demonstrate a linear correlation between the reaction energies (ΔE) and E_a_ (Figure 1a), indicating the well-known Brønsted–Evans–Polanyi (BEP) correlation [42]. The BEP correlation makes it possible to predict the effectiveness of metal catalysts with the same mechanism for activating reagents and, therefore, with the same surface geometry of the resulting transition complex. It is important to note that the dissociation of water is accompanied by a higher activation energy than the hydride dissociation (Figure 1a). The high contribution of water activation to the RDS of AB hydrolysis can be explained by the stronger O-H bond (429.9 kJ/mol) in water than the B-N bond (377.9 kJ/mol) and B-H bond (345.2 kJ/mol) in AB [43]. The published data on DFT results confirm that activating water on a metal surface requires overcoming a higher energy barrier compared to the hydride activation stage [28,29,30].

It was additionally shown that there is a relationship between E_ads_ and E_a_ for breaking the O-H bond in the water molecule (Figure 1b). This is consistent with the Sabatier principle [44], which states that the adsorption energy of water molecules on the catalyst surface should be strong enough to allow for easier water dissociation. At the same time, the adsorption energies of the intermediates (OH*, H*) should be optimal to ensure their further transformation with the release of active centers for further adsorption of reagents. This relationship is common in DFT calculations for water dissociation. The comparison of the DFT results of this work with literature data for different metal surfaces is presented in Figure S3a.

#### 3.1.2. Activity of Co Metal Catalyst in the Hydrolysis of NaBH_4_, NH_3_BH_3_, and (CH_2_NH_2_BH_3_)_2_

**Figure 2:**
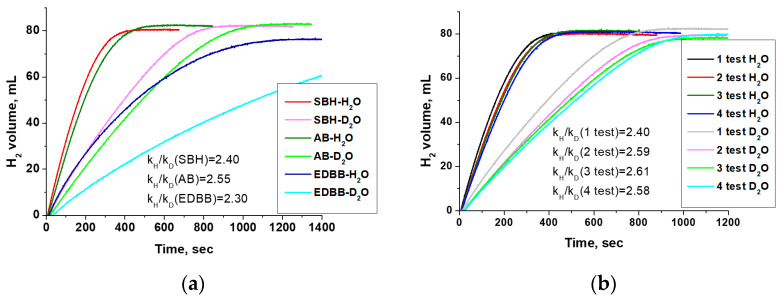
(**a**) The effect of hydride nature and replacement of H_2_O with D_2_O on the rate of H_2_ evolution in the presence of the Co^0^ catalyst. (**b**) The calculated k_H_/k_D_ ratios at cycling durability tests with the Co^0^ catalyst in NaBH_4_ hydrolysis.

The DFT results presented above predict higher cobalt activity in chemical processes proceeding with the water dissociation stage (5). This is confirmed by numerous published experimental data on the high catalytic activity of the cobalt-containing catalysts in the hydrolysis reaction of both NaBH_4_ and NH_3_BH_3_ [4,5,23,45]. The activity of cobalt catalysts in hydrolysis (CH_2_NH_2_BH_3_)_2_ has yet to be well studied, but according to our research, the Co^0^ catalyst synthesized by galvanic replacement is quite active in this process. The rate of hydrogen release is consistent with the reducing ability of the hydride and decreases in a row: SBH > AB > EDBB (Figure 2a). This appears to be due to the difference in hydride activation on the catalyst surface.

The KIE measurement using isotopically labeled reagents is a widely used experimental technique to reveal the RDS. The results on KIE for catalysts containing metal active components have mostly been investigated in processes (1) and (2). They have shown that for the hydrolysis of both SBH and AB, a primary isotopic effect (k_H_/k_D_ > 2) is observed when the reaction takes place in deuterated water, and a secondary KIE is noted with isotope-labeled hydrides [43,46,47]. Our tests of the Co^0^ catalyst in heavy water are shown in Figure 2a. As expected, there is a slowdown in the hydrogen evolution rate. For all studied hydrides, k_H_/k_D_ > 2 (shown in Figure 2a) is obtained, indicating the direct formation of the deuterium bond in the structure of the activated complex. This observation supports the idea that the dissociation of water (5) can be considered as the RDS when using metallic cobalt as a catalyst. This is consistent with the DFT results and the literature data for other metal catalysts (Table S1).

The results on SBH hydrolysis show that the tested Co^0^ catalyst is quite stable. In four consecutive reactions, without separating the reaction product from the catalyst, the H_2_ generation rate remained almost constant despite the accumulation of sodium borate in the reaction medium. Also, changes in k_H_/k_D_ ratios were no more than 10 rel% (Figure 2b).

### 3.2. Features of Co_3_O_4_ Catalyst Use

#### 3.2.1. Catalytic Activity of Co_3_O_4_ in the Hydrolysis of NaBH_4_, NH_3_BH_3_, and (CH_2_NH_2_BH_3_)_2_

**Figure 3:**
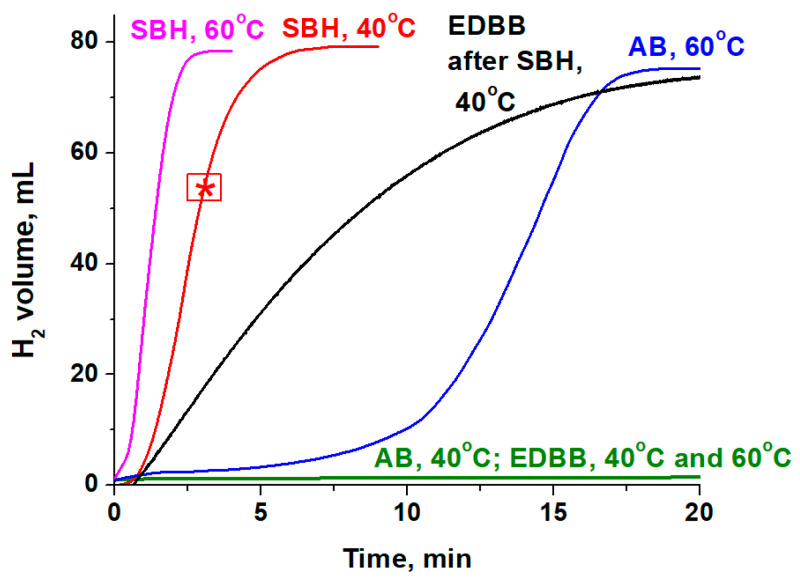
The activity of Co_3_O_4_ in the hydrolysis of studied boron-containing hydrides at 40 and 60 °C. *—sample extraction from the reaction medium for study.

It is known that Co_3_O_4_ is activated in a hydride reaction medium, and in situ, the formed active phase begins to catalyze the hydrolysis process of hydrides [23]. Our results show that at 40 °C, hydrogen evolution occurs only during SBH hydrolysis after a short induction period (~1 min) (Figure 3). When the reaction temperature rises to 60 °C after a sufficiently long induction period (~10 min), active hydrogen release begins for AB hydrolysis. It was previously shown [17] that the duration of the induction period on the hydrogen generation curve depends on the reduction rate of cobalt oxide.

So, the Co_3_O_4_ reduction or activation process is rapid in the reaction medium of a stronger reducing agent such as SBH, providing a short induction period and a high H_2_ generation rate. Unlike SBH and AB, H_2_ evolution from EDBB solution was not observed even 60 min later at 60 °C (Figure 3), demonstrating the difficulty in the formation of active phase from Co_3_O_4_ under these conditions. On the other hand, the preactivation of Co_3_O_4_ under the SBH hydrolysis allowed EDBB to be hydrolyzed at 40 °C during the next addition of EDBB to the reactor. Thus, in the row of SBH < AB < EDBB, the duration of the induction period is increased. This is a result of a decrease in the reductive ability of the studied boron-containing hydrides toward Co_3_O_4_.

**Figure 4:**
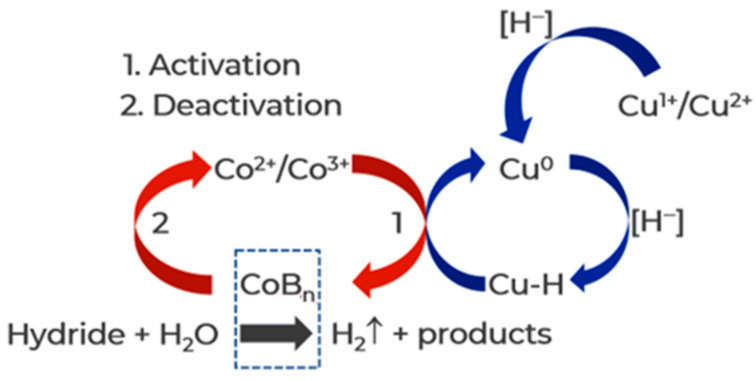
The proposed scheme of Co_3_O_4_ activation by copper compounds in the reaction medium of hydrides.

It is established that modifying cobalt oxide-based catalytic systems with copper enhances their activation rate and activity in the reaction medium of AB hydrolysis, as documented in references [48,49,50,51,52]. When discussing these results, the following explanation is offered (Figure 4): Since metals with high reduction potential are more easily reduced by hydride, Cu^2+^ (0.337 V) is first reduced to Cu^0^ in the reaction media, followed by the formation of surface Cu-H centers. The high reducing ability of these centers facilitates the easier reduction in cobalt in oxide compounds. We think that the enhanced durability of the catalysts is also a result of this mechanism. Cu-H species may prevent oxidation of cobalt active component in the reaction medium.

**Figure 5:**
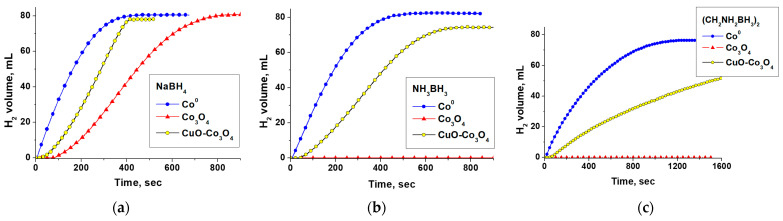
The comparison of activity of Co^0^, Co_3_O_4_, and CuO (10 wt%)-Co_3_O_4_ in the hydrolysis of (**a**) NaBH_4_, (**b**) NH_3_BH_3_, and (**c**) (CH_2_NH_2_BH_3_)_2_ at 40 °C.

Therefore, to improve the activation and increase the activity of Co_3_O_4_ in AB and EDBB hydrolysis at 40 °C, Co_3_O_4_ has been modified by a small amount of CuO (10 wt%). To do this, Co_3_O_4_ was ground with CuCO_3_·Cu(OH)_2_ in a hand mortar and calcined at a low temperature (300 °C) to decompose the basic copper carbonate to CuO and maintain Co_3_O_4_ dispersity. The XRD analysis confirmed that the sample was a mixture of two crystalline phases, 9% CuO + 91% Co_3_O_4_. The average size of Co_3_O_4_ crystallites (CSR) remained unchanged (41 ± 2 nm). As expected, the application of the CuO-Co_3_O_4_ catalyst in SBH hydrolysis results in a significant decrease in the induction period as well as an increase in the rate of hydrogen generation, as Figure 5a illustrates. Almost instantaneous hydrogen generation from aqueous solutions of AB and EDBB is also observed. (Figure 5b,c). The activity differences between Co^0^, Co_3_O_4_, and CuO-Co_3_O_4_ are thought to be explained by the various types and contents of catalytically active centers involved in the activation of reagents in the reaction medium.

As mentioned earlier, previous studies have already investigated the reduced forms of cobalt (CoB_n_) that are produced in situ from cobalt precursors and play a crucial role in catalyzing the studied processes [17,23,24,25]. However, the main focus of this study is on the amount and state of the remaining Co_3_O_4_ phase and its influence on the results of KIE measurements and DFT modeling of the water activation stage.

#### 3.2.2. Study of Co_3_O_4_ Activated in the Reaction Medium of NaBH_4_

In order to estimate the reduction degree of Co_3_O_4_ and study its characteristics, during the stage of vigorous H_2_ generation, the sample of activated oxide was removed from the reaction medium of NaBH_4_ hydrolysis at 40 °C after 3 min by a magnet (Figure 3). The sample was then subjected to XRD examination while being analyzed under an alcohol layer to avoid its oxidation by air. The results (Table 2) indicate that the Co_3_O_4_ structure is mainly preserved and that the CoO phase content is about 5 wt%.

**Figure 6:**
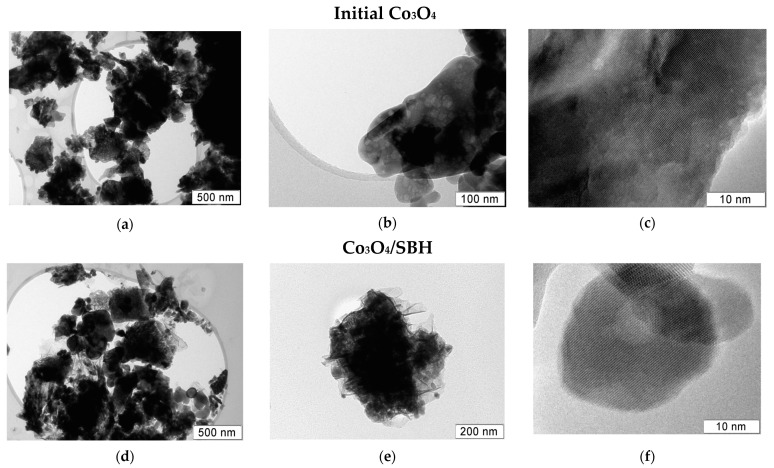
The TEM images of different magnitudes for initial Co_3_O_4_ (**a**–**c**) and Co_3_O_4_ extracted from the reaction medium of NaBH_4_ (**d**–**f**).

The study of the activated Co_3_O_4_/SBH was continued with TEM (Figure 6). Before the study, the sample was dried in a vacuum and stored under Ar. TEM revealed that this sample largely maintains the original oxide’s morphology (Figure 6a,b,d,e). However, the surface of the oxide particles has been altered by the action of hydride. The formation of translucent films and contrast rounded particles of amorphous Co_x_B particles is predictably detected (Figure 6d–f). Nanosized Co_x_B particles are observed in transparent films (Figure 6d,e) and on the surface of Co_3_O_4_ (Figure 6f). The formation of the active cobalt boride phase has been discussed previously, for example, in [17]. However, in this study, we were primarily interested in the oxide phase in activated Co_3_O_4_. For this purpose, TPR with H_2_, Raman, and ATR FTIR spectroscopy were further applied.

**Figure 7:**
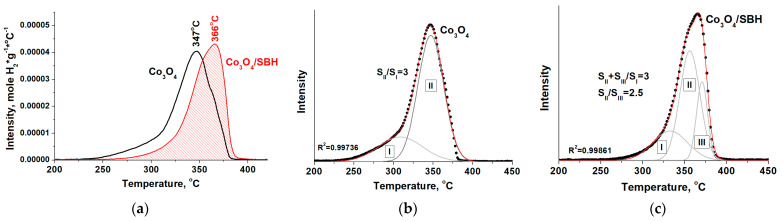
(**a**) TPR H_2_ spectra for initial Co_3_O_4_ and after its treatment in NaBH_4_ solution (Co_3_O_4_/SBH), deconvolution of spectra of initial Co_3_O_4_, (**b**) and Co_3_O_4_/SBH (**c**).

Note that in the TPR H_2_ study, the sample briefly came into contact with air at the stage of preparation of the experiment (weighing and loading into the reactor). Figure 7 shows that after the activation of Co_3_O_4_ in the sodium borohydride solution, there is a significant change in the TPR H_2_ spectrum. First of all, there is a shift of the reduction process to high temperatures at 19 °C, indicating a decrease in the reduction potential of cobalt cations in its structure.

It is well known [33] that the traditional Co_3_O_4_ reduction process is in two stages: (I) the low-temperature wide peak corresponds to the reduction phase of Co^3+^ to Co^2+^, and (II) the high-temperature narrow peak corresponds to the reduction of Co^2+^ to Co^0^ (Figure 7b). For the Co_3_O_4_ stoichiometry, the ratio of the peak area II to the peak area I must be three, which we have confirmed in our experiment for initial Co_3_O_4_. The deconvolution of the TPR H_2_ spectrum of the activated Co_3_O_4_/SBH (Figure 7c) is different. In this case, the higher temperature hydrogen consumption is described more precisely by the three peaks. Two high-temperature peaks (II and III) can be expected to be attributed to the reduction in two Co^2+^ states. For the initial Co_3_O_4_, the amount of hydrogen consumed for the reduction was 1.25 × 10^−2^ mol/g. This corresponds to the calculated Co_3_O_4_ reduction degree of 75%. The loss of oxygen during Co_3_O_4_ reductive treatment with SBH solution is low, as the amount of hydrogen consumed during reduction in this sample has decreased only by 9 rel%.

The increase in the Co_3_O_4_/SBH reduction temperature and the structural diversity found in its TPR H_2_ spectrum may be due to defects in the oxide structure. It is known that Raman spectroscopy is often used to detect defects in Co_3_O_4_. But, the Raman spectra interpretation for polydisperse Co_3_O_4_ is problematic. When analyzing them, the dimensional effects [53], degree of crystallinity [54,55], particle aggregation [56], and defects [57,58] should be taken into account. All these parameters affect the position of the peaks and their half-width. Unfortunately, when analyzing Raman spectra of Co_3_O_4_ with structural defects, in most published works, the influence of other factors is not taken into account. This appears to be a reason for numerous contradicting reports found in the literature regarding the relationship between the formation of anionic vacancies in the structure of Co_3_O_4_ and the resulting change in its Raman spectrum. There is information that this process may be accompanied by both the red shift of the A_1g_ peak [33,58,59] and its blue shift [60,61]. In this case, the frequently mentioned phonon confinement effect should be applied to nanoscale samples (<10 nm) [62].

**Figure 8:**
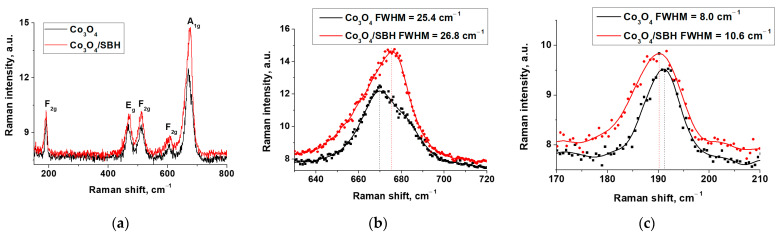
Raman spectra for Co_3_O_4_ and Co_3_O_4_/SBH (**a**), A_1g_ peak region (**b**), and F_2g_ peak region (**c**).

The obtained spectrum of initial Co_3_O_4_ has five peaks (Figure 8) at 670, 606, 513, 468, and 191 cm^−1^. These correspond to A_1g_, F_2g_, F_2g_, E_g_, and F_2g_ vibrations, respectively [63]. The peak of A_1g_ relates to vibrations of Co^3+^-O in the octahedron, and the peak of F_2g_ relates to vibrations of Co^2+^-O in the tetrahedron. Note that our preliminary study of the Co_3_O_4_/SBH sample by TPR H_2_ has shown that the reducing treatment of Co_3_O_4_ with SBH only slightly reduced the oxygen content, resulting in structural diversity of the cobalt oxygen environment and a stronger Co-O bond. XRD analysis showed that the Co_3_O_4_ crystalline phase characteristics in the Co_3_O_4_/SBH did not change, which eliminates the influence of the Co_3_O_4_ crystal size on the Raman peak position. However, the appearance of magnetic properties in the Co_3_O_4_/SBH sample due to the formation of the ferromagnetic amorphous phase of cobalt boride [17] can increase the degree of aggregation of particles, which, above all, should lead to a shift of A_1g_ to the low-frequency region.

On the other hand, Raman spectra analysis of Co_3_O_4_/SBH shows that the A_1g_ peak is shifted into the high-frequency region at 6 cm^−1^. The shape of the A_1g_ peak and its full width at half maximum (FWHM) also change (Figure 8b). We believe that the observed asymmetry, increased FWHM, and blue shift of A_1g_ are due to the strengthening of Co-O bonds, which is a result of the formation of anionic vacancies in the Co_3_O_4_ structure and the redistribution of electron density. Note that, according to DFT calculations, the most likely oxygen removal is from the octahedral oxygen environment.

Similar results have been reported in [60], in which the blue shift of the A_1g_ peak in the Raman spectrum has been observed for the Co_3_O_4_ sample, characterized by an increasing hydrogen reduction temperature (TPR H_2_ data) and a shortening of the Co-O bond (EXAFS data). Changes in the geometry of [CoO_6_] octahedra must cause geometry changes in adjacent [CoO_4_] tetrahedra (Figure 8c). In fact, there is a noticeable broadening and shift to the low-frequency region for the F_2g_ peak in the Raman spectrum of Co_3_O_4_/SBH, which might correspond to an elongation of the Co-O bonds in tetrahedra.

**Figure 9:**
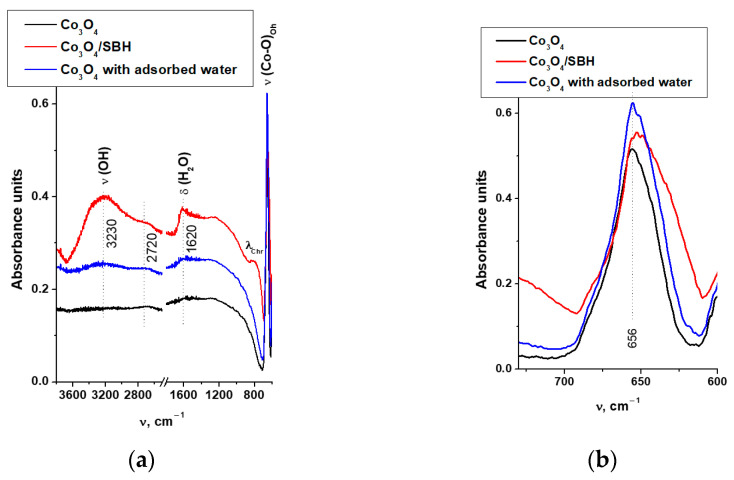
(**a**) ATR FTIR spectra of initial Co_3_O_4_ and Co_3_O_4_/SBH, as well as initial Co_3_O_4_ with preadsorbed water on its surface, and (**b**) Co-O vibration region.

The comparative study of Co_3_O_4_ and Co_3_O_4_/SBH was continued with ATR FTIR spectroscopy. Figure 9 shows the spectra of initial Co_3_O_4_ and Co_3_O_4_/SBH, as well as initial Co_3_O_4_, on the surface of which water was preadsorbed. Adsorption was carried out in an equilibrium water-vapor system at room temperature for 20 h. It is evident that the Christiansen effect is present in all spectra. It appears at wavelengths (λ_Chr_) when the refractive indices of the matrix (air) and the sample (Co_3_O_4_) are equal. It can be observed when the bulk of the sample consists of particles that are evidently larger than the wavelength of the irradiating light (2–25 μm). It agrees with the SEM data of the studied Co_3_O_4_ (Figure S1a–c).

Figure 9a shows that the spectra of Co_3_O_4_ and Co_3_O_4_/SBH samples differ in the intensity of the broad absorption bands (a. b.) with maximums at 3230 and 2720 cm^−1^ corresponding to the hydroxyl groups’ valence vibration and at 1620 cm^−1^ corresponding to the deformation vibration of adsorbed water molecules. These a. b. are more intensive in the spectrum of Co_3_O_4_/SBH. This indicates it is impossible to thoroughly dehydrate the sample after treating Co_3_O_4_ in SBH solution, washing, and vacuum drying for a long period of time at room temperature. In the spectrum of initial Co_3_O_4_, these a. b. are barely visible. The long-time saturation of the surface of initial Co_3_O_4_ with water vapor increased the intensity of the discussed absorption bands. However, the intensity achieved is significantly lower than the intensity of vibrations of water and hydroxyl groups in the spectrum of Co_3_O_4_/SBH. This suggests that the high water content on the surface of the Co_3_O_4_/SBH sample is due to its stabilization at the anionic vacancies. In addition, the increased background absorption in the Co_3_O_4_/SBH spectrum indicates the presence of free charge carriers (CoB_n_) and the possible formation of proton conductivity by involving hydroxyl groups in the hydrogen bond.

A more detailed analysis of the a. b. of stretching vibration of Co-O bonds in the octahedral environment with the maximum at 656 cm^−1^ (Figure 9b) reveals its broadening on the low-frequency slope and a slight low-frequency shift of the maximum at 3 cm^−1^ (red shift). This suggests that adsorbed water weakens Co-O bonds.

The observation of different types of a. b. shifts corresponding to those for [CoO_6_] observed by Raman spectroscopy (blue shift) and ATR FTIR spectroscopy (red shift) is related to different conditions for receiving spectra. In the case of Raman spectroscopy, it is known that the surface of the sample is heated by the laser, resulting in adsorbed water being removed from the surface, which leads to a stronger Co-O bond. In the case of ATR FTIR spectroscopy, there is no heating of the sample, allowing us to obtain information about the Co_3_O_4_ surface containing adsorbed water on defects.

Thus, the TPR H_2_ study shows that the reduction degree of Co_3_O_4_ in the reaction medium of NaBH_4_ is not significant. XRD analysis confirms that Co_3_O_4_ remains the main crystalline phase. However, the shift of the oxide phase reduction process to higher temperatures indicates an increase in the electronic density of cobalt atoms and the formation of oxygen vacancies. These results are in accordance with the data of [31,32,33,34,35], where the Co_3_O_4_ samples treated with NaBH_4_ solution were examined by a set of methods such as XRD, XPS, EPR, TPR H_2_, TPD O_2_, etc. On the other hand, the vibration spectroscopy in this work confirms the differences in the structure of the nearest cobalt environment for Co_3_O_4_ and Co_3_O_4_/SBH. It is important to note that the formation of anionic vacancies on the Co_3_O_4_/SBH surface increases the amount of adsorbed water, which is difficult to remove from the surface under long vacuum drying conditions at room temperature.

#### 3.2.3. Modeling of H_2_O Adsorption on the Surface of Co_3_O_4_ and Co_3_O_4_ with Oxygen Vacancies

Numerous publications have traditionally reported the formation of anionic vacancies in the structure of transition metal oxides and a decrease in the oxidation state of metals during the hydrolysis tests of SBH and AB [22,31,64,65]. However, the discussion of the role of Co_3_O_4_, including defective ones, in the water activation stage (5) is presented in single publications [36,37,38].

**Figure 10:**
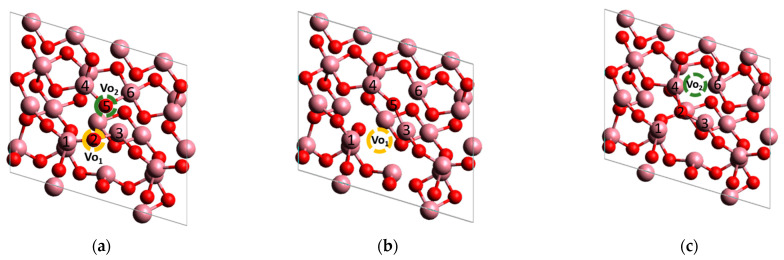
(**a**) The optimized structure of initial 311 Co_3_O_4_ clusters and Co_3_O_4_ clusters with oxygen vacancies (Vo_1_ (**b**) and Vo_2_ (**c**)). The atoms of Co are pink, and O are red.

In order to study the structural changes in Co_3_O_4_ during the formation of anionic vacancies, DFT calculations were performed. When studying the 311 surface of Co_3_O_4_ (Fm3m), two positions for oxygen vacancies were considered (Figure 10): Co^3+^-O-Co^2+^ (Vo_1_ –yellow) and Co^3+^-O-Co^3+^ (Vo_2_-green). Our calculations showed that the formation of Vo_1_ required more energy (2.65 eV) than the formation of Vo_2_ (2.10 eV). This suggests that anionic vacancies are more likely to be in an octahedral Co^3+^ environment than in a tetrahedral Co^2+^ environment.

The optimization of oxide cluster structures has shown that vacancies cause shorter Co-O lengths in the cobalt octahedron environment near the vacancy. When Vo_1_ and Vo_2_ appear, the total length of Co-O bonds near the vacancy decreases by 1.8 and 4.7 rel%, respectively, which confirms the blue shift of A_1g_ in the Raman spectrum of Co_3_O_4_/SBH (Figure 8b). It is noted that the oxygen atom 2 is moved to the octahedron near the vacancy after the creation of the Vo_2_ vacancy (Figure 10c). This results in a 3.62 rel% increase in Co-O bond lengths in the nearest tetrahedron and is consistent with the results of Raman spectroscopy on red shift of F_2g_ in the spectrum of Co_3_O_4_/SBH (Figure 8c).

**Figure 11:**
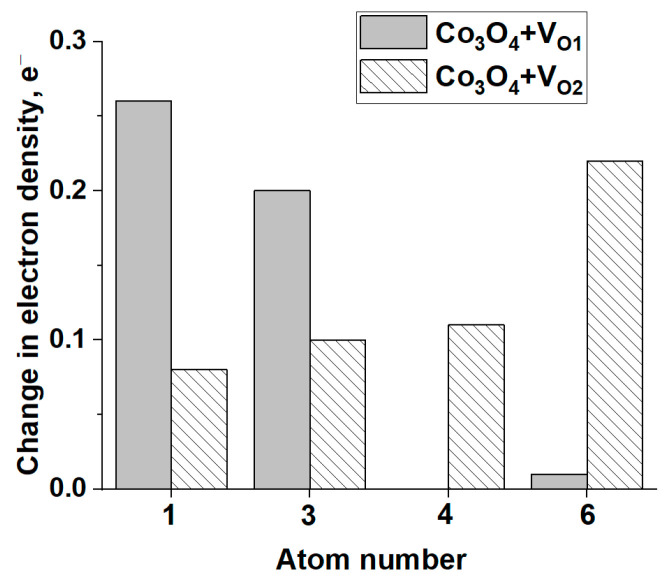
Changes in the electron density of cobalt atoms relative to Co_3_O_4_. The numbers of the Co atoms correspond to the atoms in Figure 10.

As expected, the formation of anionic vacancies in the Co_3_O_4_ structure leads to an increase in the electron density on the Co atoms adjacent to the vacancy (Figure 11), which reduces its oxidation state. A similar result was discussed in [66]. As a result, the mobility of lattice oxygen is decreased, which explains the observed increase in the reduction temperature in the TPR H_2_ spectrum of Co_3_O_4_/SBH (Figure 7a), as in the TPR H_2_ spectra of other similar samples [33,60].

**Figure 12:**
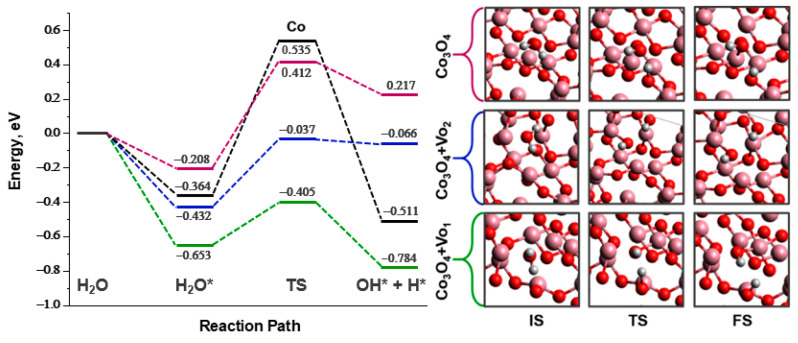
Energy profile of dissociative adsorption of water on the surfaces of Co_3_O_4_ and Co_3_O_4_ with oxygen vacancies with structures of initial (IS), transition (TS), and final (FS) states. The atoms of Co are pink, O are red, and H are grey.

Calculations have confirmed that creating anionic vacancies and increasing the electron density on cobalt atoms enhances the adsorption of water molecules. The adsorption energy decreases from −0.208 eV for Co_3_O_4_ to −0.653 eV and −0.432 eV for Co_3_O_4_ + Vo_1_ and Co_3_O_4_ + Vo_2_, respectively. Oxygen vacancies also positively affect the stage of activation of water. The activation energy of O-H breakdown on the Co_3_O_4_ surface with vacancies Vo_1_ (0.248 eV) and Vo_2_ (0.395 eV) is less than that in the case of initial Co_3_O_4_ (0.620 eV) (Figure 12). These results are consistent with the literature [37,38]. It is shown that the OH* group formed during the dissociation of water occupies the position of absent lattice oxygen if this is sterically available. At the same time, there is an increase in the total length of Co-O bonds by 0.99 rel% and 0.11 rel% near Vo_1_ and Vo_2_, respectively. This corresponds to the results of ATR FTIR spectroscopy (Figure 9) on the weakening of Co-O bonds in [CoO_6_] in the case of the Co_3_O_4_/SBH containing the adsorbed water.

**Figure 13:**
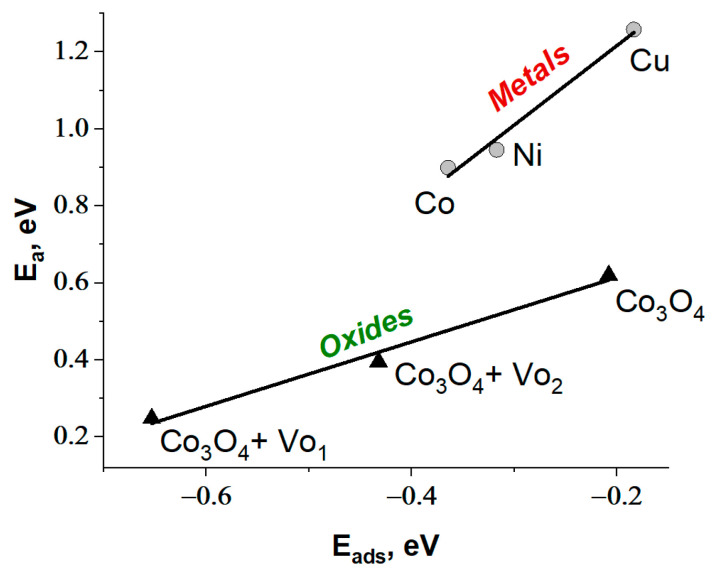
Comparison of linear correlations between E_ads_ and E_a_ (the Sabatier principle) for the dissociative adsorption of H_2_O on modeled surfaces of cobalt oxide and metals.

Similar to metals (Figure 1b), DFT calculations on cobalt oxide show a decrease in activation energy with a reduction in adsorption energy. There is a linear correlation between these values (Figure 13): the lower the E_ads_ of water, the lower the E_a_ for the O-H bond dissociation. For instance, similar conclusions were obtained in [64] to explain the high activity of Cu^0^/Cu_0.76_Co_2.24_O_4−δ_ in AB hydrolysis. It should be mentioned that the linear E_a_-E_ads_ relationships for metals and oxides do not coincide (Figure 13). Metals have higher activation energy values. These results correspond with known statements that the dissociation of water on the polarized oxide surface occurs with lower activation energies than on metal surfaces [36,67]. The comparison of the DFT results of this work with literature data for different metal oxide surfaces is presented in Figure S3b.

Thus, based on the published literature and the results of this study, it can be concluded that the high-activity state of the cobalt oxide catalyst can be considered as CoB_n_ active centers immobilized in the oxide matrix with anionic vacancies (Co_3_O_4−δ_). It can be assumed that the oxide phase serves not only as a precursor and support for the metal or metalloid cobalt active component formed during the reduction process but also as a key catalyst component that improves the water activation process. If so, this suggests that when H_2_O is replaced with D_2_O in the catalytic hydrolysis of the boron-containing hydrides, a Co_3_O_4_-based catalyst is to be characterized with a different kinetic isotope effect compared to a metal catalyst.

#### 3.2.4. Comparison of Kinetic Isotope Effect Results for Co_3_O_4_

**Figure 14:**
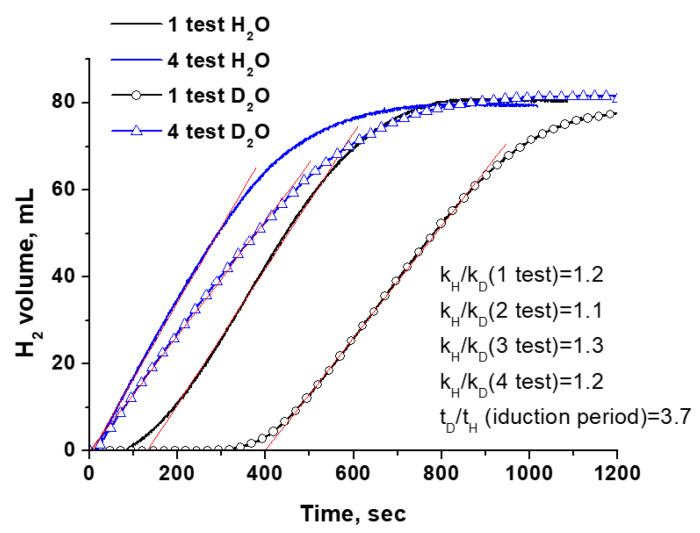
Change in kinetics of H_2_ generation over Co_3_O_4_ in NaBH_4_ hydrolysis at replacement H_2_O by D_2_O (40 °C).

It should be noted that the kinetic isotope effect (KIE) in the hydrolysis of boron-containing hydrides measured at replacing H_2_O with D_2_O for transition metal oxide-based catalysts is almost nonpublished in the literature. The results obtained in our work on the KIE in SBH hydrolysis in the presence of Co_3_O_4_ are presented in Figure 14.

Four successive reaction cycles were carried out. First of all, it is evident that the use of D_2_O almost four times lengthens the induction period. It indicates that D_2_O is directly involved as a reagent when reducing oxide in a reaction medium takes place. When calculating the KIE, the induction period was not taken into account. Only hydrogen evolution kinetics after the activation stage was analyzed. The k_H_/k_D_ values for four consecutive tests are shown in Figure 14. It can be seen that the H_2_ generation rate in the presence of Co_3_O_4_ is only slightly reduced when using D_2_O. The k_H_/k_D_ value is changed little during cyclic testing, indicating the relative stability of the active component in the activated oxide catalyst. It can be assumed that the reduction degree of Co_3_O_4_ changes little after the activation stage.

This result is consistent with the pioneer study published in 2022 [68]. In this work, nickel and cobalt oxides have been shown to have a relatively low reduction rate in the reaction medium since H* formed by the dissociation of B-H, after forming the catalytically active cents on activation reagents in sufficient quantity, is not spent on oxide reduction and reacts mainly with H*, formed during water dissociation, with hydrogen release.

Similar results were obtained in the hydrolysis of AB and EDBB over Co_3_O_4_ modified by CuO (Figure S4). Table 3 shows that when Co_3_O_4_ is tested in SBH hydrolysis as well as 10% CuO-90% Co_3_O_4_ in AB and EDBB hydrolysis, k_H_/k_D_ < 2 (the secondary kinetic isotope effect) is obtained. Note that in the experiments with the metallic Co^0^ catalyst, the primary isotope effect has been fixed (k_H_/k_D_ > 2; see Section 3.1.2).

Thus, the activation of water on the in situ-activated Co_3_O_4_ catalyst surface is facilitated. This is confirmed not only by KIE measurements but also by DFT modeling. Based on the discussions mentioned earlier, activated Co_3_O_4_ catalysts should be considered as nanosized CoB_n_ immobilized in the anion-deficient Co_3_O_4−δ_. If the Co_3_O_4−δ_ phase was not involved in the water activation process, the k_H_/k_D_ values would be more than two.

## 4. Conclusions

In the hydrolysis processes of boron-containing hydrides like SBH and AB, the roles of Co_3_O_4_ and Co^0^ have previously been examined, with these findings discussed in the introduction. The new study of them is explained by the need to clarify their catalytic properties from the modern position of a bifunctional catalyst with centers activating hydride and water. It was also interesting to consider the results for SBH and AB hydrolysis together with the results for EDBB hydrolysis, where the use of such catalysts had not yet been described.

DFT calculations for the dissociative adsorption of water and AB on metal clusters Co, Ni, and Cu showed an implementation of the Brønsted–Evans–Polanyi principle and confirmed that the water activation stage is characterized by a higher activation energy. For this stage, a linear correlation between E_ads_ and E_a_ (the Sabatier principle) was found among the metals studied. Cobalt has the lowest E_ads_ and E_a_, which is supported by numerous literature data on the high activity of cobalt-containing active components. The study of the kinetic isotope effect by replacing H_2_O with D_2_O confirmed that in the case of Co^0^, the primary isotopic effect is found, i.e., the breaking of the OH-bond in the water molecule determines the rate of hydrogen generation and can be considered as a rate-determining stage.

It is known that Co_3_O_4_ is activated in a reaction medium of hydrides, and the in situ formed active phase begins to catalyze the hydrolysis process of boron-containing hydrides. It was shown that in the row of SBH > AB > EDBB, Co_3_O_4_ activity decreases. This is a result of a decrease in the reductive ability of the studied boron-containing hydrides toward Co_3_O_4_. In the case of AB and EDBB hydrolysis, this problem can be solved by adding copper compounds to the cobalt oxide catalysts. According to our study (TEM, TPR H_2_, Raman, and ATR FTIR spectroscopy), the reduction degree of Co_3_O_4_ in the reaction medium of SBH with a high reducing ability is low, and the active state of the oxide catalyst providing a high H_2_ generation rate should be considered as nanodispersed CoB_n_ immobilized in the anion-deficient Co_3_O_4−δ_. According to ATR FTIR spectroscopy, the presence of defects on the surface of Co_3_O_4_ enhances water adsorption.

DFT modeling shows that the formation of an anion-deficient structure of Co_3_O_4_ leads to an increase in the electron density on Co atoms near the vacancies. The presence of anionic vacancies on the oxide surface facilitates the adsorption and activation of water. The localization of oxygen defects in the oxide structure determines the values of E_ads_ and E_a_. According to the Sabatier principle, these parameters are also related to a linear dependence. As expected, the E_a_ value for the dissociative adsorption of water on the oxide surface is significantly lower than on the metal surface. KIE measurements for Co_3_O_4_ in SBH hydrolysis and 10% CuO-90% Co_3_O_4_ in SBH, AB, and EDBB hydrolysis reveal a secondary KIE, indicating that the activation of water is not part of RDS. The data obtained support the statement that the creation of anionic vacancies in the structure of catalysts is a standard technique to improve the transformation of reagents containing an electron-donor atom.

Although the activity of the Co^0^ catalyst depends on water activation, the hydrogen generation rate is high. We believe this may be attributed to the relatively easier process of hydride activation. To enhance the water activation processes, we propose the addition of activating water oxide to the catalytic composition containing Co^0^. Oxides can serve not only as supports but also as water activation components. When developing a catalyst, finding the optimum proportion of metal/metalloid to oxide is crucial. This allows one to not only stabilize and modify the active metal-like component but also introduce the requisite number of water activation centers.

## Figures and Tables

**Table 1:** Adsorption energy values (E_ads_) for H_2_O and NH_3_BH_3_ and corresponding changes in the geometry of adsorbed molecules calculated in this work.

Metal	E_ads_ H_2_O, eV	M-O, Å	∠H-O-H, °	E_ads_ AB, eV	M-H, Å	∠H-B-H, °
Co	−0.364	2.200	105.7	−2.451	1.162	142.7
Ni	−0.317	2.167	105.6	−1.864	1.594	137.2
Cu	−0.184	2.349	105.0	−1.233	1.814	115.6
H_2_O/NH_3_BH_3_			104.4			113.4

**Table 2:** XRD data for initial sample Co_3_O_4_ and Co_3_O_4_ sample activated in the reaction medium of NaBH_4_ hydrolysis.

Sample	Phase Composition, wt%	SCR ^1^, nm	a(Co_3_O_4_) ^2^, Å
Co_3_O_4_	100% Co_3_O_4_	41	8.084 (±0.001)
Co_3_O_4_/SBH	95% Co_3_O_4_	40	8.085 (±0.001)
	5% CoO ^3^	17	

^1^ The average size of crystallites (CSR) was calculated using the Sherrer formula for 311 reflections for Co_3_O_4_ and 111 for CoO. ^2^ a(Co_3_O_4_)—lattice parameter of Co_3_O_4_ (sp. gr. Fd3m). ^3^ The oxidation of CoO takes place when the protective alcohol layer evaporates and the sample comes into contact with air.

**Table 3:** Kinetic isotope effect (k_H_/k_D_) for the cobalt-based catalysts in the catalytic hydrolysis of boron-containing hydrides at 40 °C (first test).

Sample	NaBH_4_	NH_3_BH_3_	(CH_2_NH_2_BH_3_)_2_
Co^0^	2.4	2.5	2.3
Co_3_O_4_ ^1^	1.2	-	-
10% CuO-90% Co_3_O_4_	1.7	1.6	1.7

^1^ Kinetic data after the activation period were analyzed.

## Data Availability

Data are contained within the article and Supplementary Materials.

## Supplementary Materials

The following supporting information can be downloaded at: https://www.mdpi.com/article/10.3390/ma17081794/s1, Description of ammonia borane and ethylenediamine bisborane synthesis; Table S1: Kinetic isotope effect (KIE) in AB hydrolysis when replacing H_2_O by D_2_O; Figure S1: SEM images of studied catalytic materials: (a–c) Co_3_O_4_, (d–f) 10%CuO-90%Co_3_O_4_, and (g–i) Co^0^; Figure S2: Example of optimized structures of initial and final states for dissociative adsorption of (a,b) NH_3_BH_3_ (4) and (c,d) H_2_O (5) on 111 Co^0^. The atoms of Co are pink, B are peach, N are blue, H are grey, and O are red; Figure S3: For metals (a) and oxides (b), comparison of the energetic parameters (E_a_, E_ads_) calculated by DFT in this work with the literature data; Figure S4: Experimental curves of H_2_ evolution for hydrolysis of NaBH_4_, NH_3_BH_3_, and (CH_2_NH_2_BH_3_)_2_ measured at replacement of H_2_O by D_2_O over 10% CuO-90% Co_3_O_4_ catalyst at 40 °C [26,36,40,43,46,47,64,69,70,71,72,73,74,75,76,77,78,79,80,81,82,83,84,85,86,87,88,89].
