# Supplementary material for: Co and Co3O4 in the Hydrolysis of Boron-Containing Hydrides: H2O Activation on the Metal and Oxide Active Centers
Source: Materials (Basel). 2024 Apr 13;17(8):1794. doi: 10.3390/ma17081794 (PMC11050988; doi:10.3390/ma17081794)
Supplement: Supplementary file 1 [file materials-17-01794-s001.zip › materials-2938310-supplementary.pdf]

materials

Supplementary Materials

Co and Co_3O_4 in the Hydrolysis of Boron-Containing Hydrides: H_2O Activation on the Metal and Oxide Active Centers

Vladislav R. Butenko [1], Oksana V. Komova [1,*], Valentina I. Simagina [1], Inna L. Lipatnikova [1], Anna M. Ozerova [1], Natalya A. Danilova [1,2], Vladimir A. Rogov [1,2], Galina V. Odegova [1], Olga A. Bulavchenko [1], Yuriy A. Chesalov [1] and Olga V. Netskina [1,2]

1 Boreskov Institute of Catalysis SB RAS, 5 Akademika Lavrentieva Ave., Novosibirsk 630090, Russia; vladislav.but@mail.ru (V.R.B.); chesalov@catalysis.ru (Y.A.C.);
2 Department of Natural Sciences, Novosibirsk State University, 1 Pirogova Str., Novosibirsk 630090, Russia
* Correspondence: komova@catalysis.ru; Tel.: +7-383-330-74-58

Reagents for synthesis of NH_3BH_3 and $(CH_2NH_2BH_3)_2$

The reagents used were sodium borohydride—$NaBH_4$ (98%, CAS 16940-66-2, Chemical Line, Sankt-Peterburg, Russia); ammonium sulfate—$(NH_4)_2SO_4$ (GOST 3769-78, Vecton, Sankt-Peterburg, Russia); tetrahydrofuran—C_4H_8O (CAS 109-99-9, Vecton, Sankt-Peterburg, Russia); diethyl ether—$(C_2H_5)_2O$ (TU 2600-001-43852015-10, Kuzbassorghim, Kemerovo, Russia); and ethylenediamine—$NH_2(CH_2)_2NH_2$ (99.9 %, CAS RN 107-15-3, Chemical Line, Sankt-Peterburg, Russia). Prior to use, diethyl ether (DE) and tetrahydrofurane (TGF) were purified from impurities and water.

Synthesis of NH_3BH_3

The synthesis of ammonia borane (NH_3BH_3, AB) is described in the Supplementary Material of our previous work [1]. In a typical procedure, 450 mL of C_4H_8O, 4.4 g of $(NH_4)_2SO_4$, and 2.5 g of $NaBH_4$ were placed into a three-neck flask and intense stirred for 6 h at 40 °C under a flow of Ar. The solution was filtered using a glass porous filter at a reduced pressure, and then the solvent was removed on a rotary evaporator with subsequent drying of the solid sample in a vacuum box without heating. The weight of the obtained hydride raw was 2 g.

Prior to the experiments, the raw sample was purified by its redissolution in DE, followed by filtration, evaporation, and drying. In our experiment, 1 g of AB was dissolved in 200 mL of DE. The resulting solution was stirred in a closed flask for 2 h. The nondissolved impurities were separated on a glass porous filter at a reduced pressure, and all of the solvent was evaporated on a rotary evaporator at 25 °C. Then, the solid AB was dried in a vacuum box. The yield was 80%.

To determine the ammonia borane purity, the process of its catalytic hydrolysis was carried out. The purity of the final product was 97%. The characterization of the hydride sample by XRD and ATR FTIR was shown in [2].

Synthesis of $(CH_2NH_2BH_3)_2$

For the synthesis of ethylene bisborane ($BH_3NH_2(CH_2)_2NH_2BH_3$, EDBB), a mixture of 3.4 g of AB (0.110 mol), 3.45 g of ethylenediamine (EDA, 0.052 mol) and 80 mL of TGF was heated in a 100 ml flask equipped with a condenser at 50 °C. Total dissolution of AB and ammonia gas evolution were observed. After 5 h, the temperature of the reaction mixture was slowly increased before the start of TGF boiling (66 °C). The mixture was boiled for 0.5 h; then the heating was stopped, and the hot mixture was filtered on a glass porous filter at a reduced pressure. Clouding of the solution was already observed during cooling to room temperature. TGF was evaporated with a rotary evaporator at the temperature of the bath at 40 °C. The precipitate was dried in a vacuum box without heating. The yield of EDBB raw was about 70%.

A water–isopropanol solution (50 %: 50 % by volume) was prepared and cooled to −6 °C. To clean the EDBB, 20 mL of water–isopropanol solution and 2.89 g of raw hydride sample were intensively stirred in a flask and put in an ice bath for 2 h. Further, the mixture was filtered through a glass porous filter at reduced pressure, and the precipitate was additionally washed with 30 mL of cooled water–isopropanol solution, then by 70 mL of hexane and dried in the vacuum box at room temperature. The yield of EDBB was 77%. The purity of EDBB was 96% pure according to its catalytic hydrolysis. The resulting ATR FTIR spectrum fully corresponds to the EDBB spectrum described in [3,4].

Table S1. Kinetic isotope effect (KIE) in AB hydrolysis when replacing H_2O by D_2O.

Catalyst	KIE	Reference
Co/carbon nanotubes	2.2	[5]
Co/Activated carbon	2.1	[5]
$Cu_{0.5}$–$(CoO)_{0.5}/TiO_2$	5.5	[6]
Ni_2Pt@ZIF-8	4.95	[7]
Rh nanoparticles	2.85	[8]
Pt nanoparticles	2.32	[8]
Rh nanoparticles	2.3	[9]
RuNP@NH_2-PIILS *	2.31	[10]
Au_1Ru_1@dendrimer	2.25	[11]
$Rh_{0.25\%}Co_{3\%}/TiO_2$	3.00	[12]
$Ru_{0.25\%}Co_{3\%}/TiO_2$	2.79	[12]
$Rh_{0.25\%}/TiO_2$	3.60	[12]
$Ru_{0.25\%}/TiO_2$	3.14	[12]
NiNPs/ZIF-8	2.49	[13]
Ni_2P	2.9	[14]
$Ni_{12}P_5$	2.8	[14]
Ni_3P	2.4	[14]
Pd@$Ni_{12.5}P_{40}$/rGO	3.1	[15]
Pd/O-CoP_2	3.7	[16]
Pd/CoP_2	3.6	[16]
Pt/ carbon nanotubes	1.9	[17]
$Pt_{0.25\%}Co_{3\%}/TiO_2$	2.79	[12]
$Pt_{0.25\%}/TiO_2$	3.38	[12]
Pt/Co nanoparticles	2.46	[18]
Ru/ carbon nanotubes	3.15	[19]

* $NaBH_4$ hydrolysis.

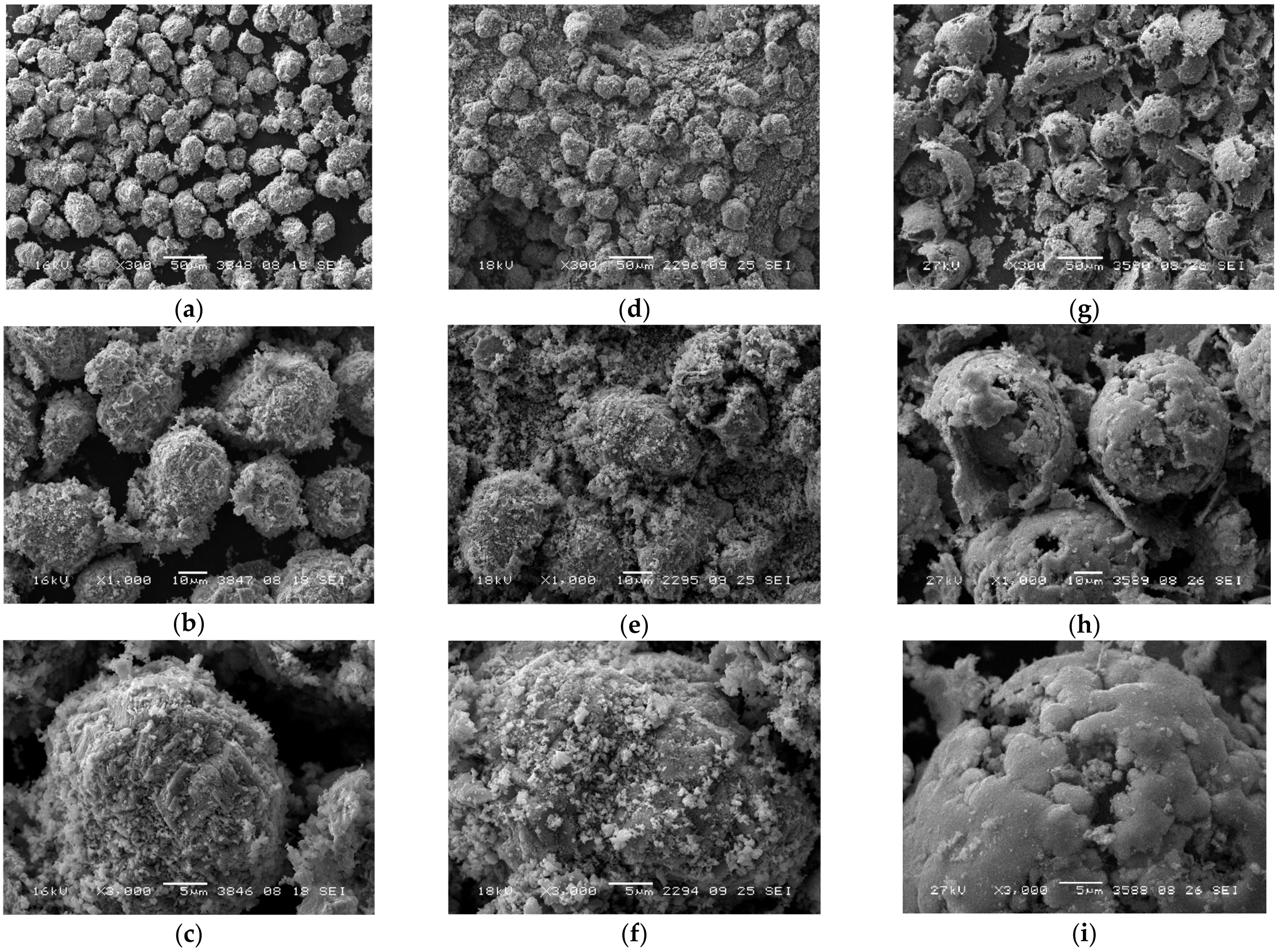

Figure S1. SEM images of studied catalytic materials: (**a**–**c**) Co_3O_4, (**d**–**f**) 10 %CuO-90 %Co_3O_4, and (**g**–**i**) Co^0.

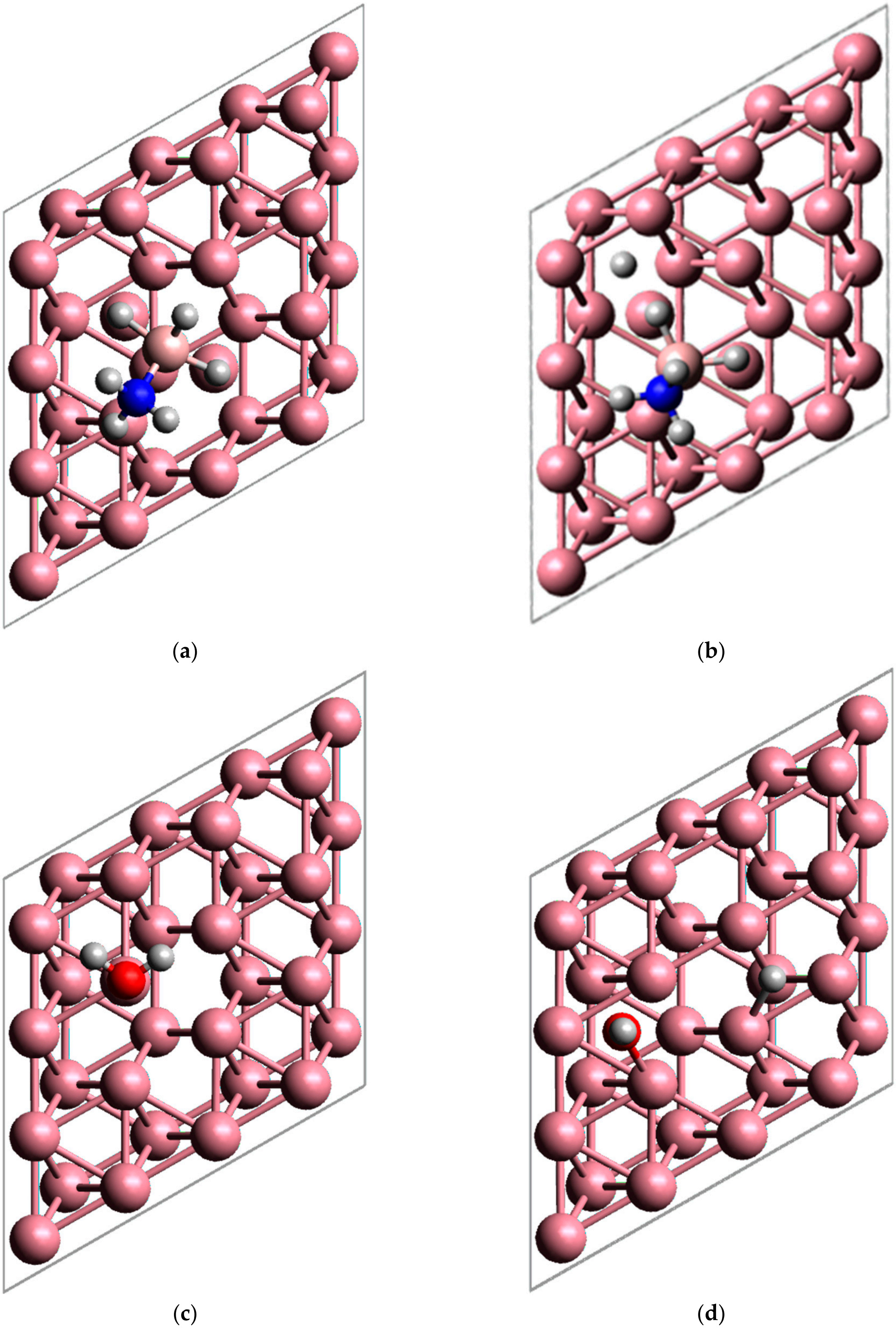

Figure S2. Example of optimized structures of initial and final states for dissociative adsorption of (**a**,**b**) NH_3BH_3 (4) and (**c**,**d**) H_2O (5) on 111 Co^0. The atoms of Co are pink, B are peach, N are blue, H are grey, and O are red.

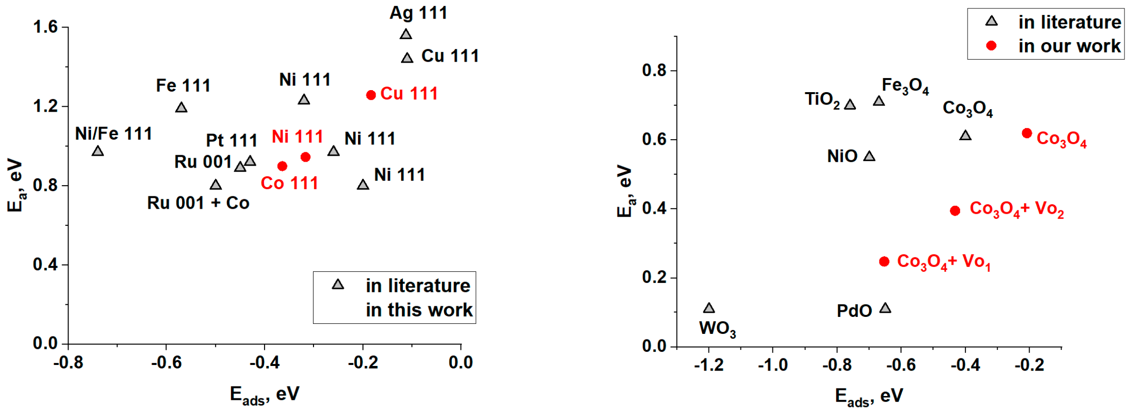

(**a**) (**b**)

Figure S3. For metals (**a**) and oxides (**b**), comparison of the energetic parameters (E_a, E_{ads}) calculated by DFT in this work with the literature data [12,19,20–28].

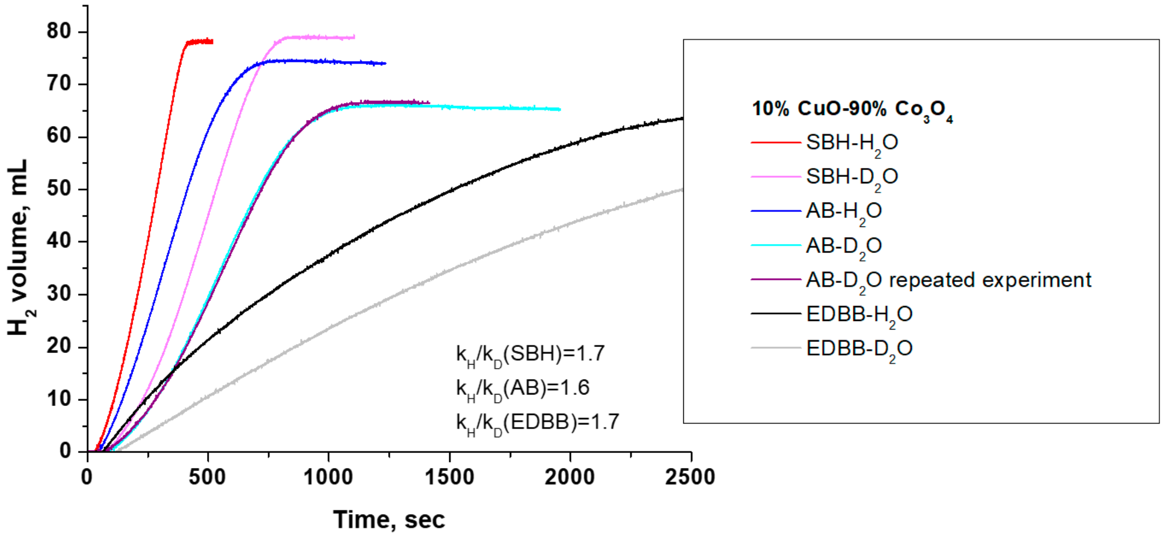

Figure S4. Experimental curves of H_2 evolution for hydrolysis of $NaBH_4$, NH_3BH_3, and $(CH_2NH_2BH_3)_2$ measured at replacement of H_2O by D_2O over 10 % CuO-90 % Co_3O_4 catalyst at 40 °C.

The obtained data could not be described within one formal kinetics. The k_H/k_D estimation was based on the assumption that substituting H_2O for D_2O would not change the reaction mechanism. Therefore, k_H/k_D may be estimated from the ratio of the reaction time, where the same hydride conversion was achieved, i.e., the same amount of hydrogen was released. Note that regardless of the hydride nature, the initial concentration of B-H bonds in the reaction medium was the same. The k_H/k_D values were averaged within the conversion of 10–80 % in 10 mL of H_2 increments.

References

1. Gorlova, A.M.; Kayl, N.L.; Komova, O.V.; Netskina, O.V.; Ozerova, A.M.; Odegova, G.V.; Bulavchenko, O.A.; Ishchenko, A.V.; Simagina, V.I. Fast Hydrogen Generation from Solid NH_3BH_3 under Moderate Heating and Supplying a Limited Quantity of $CoCl_2$ or $NiCl_2$ Solution. *Renew. Energy* **2018**, *121*, 722–729, doi:10.1016/j.renene.2018.01.089.
2. Komova, O. V.; Simagina, V.I.; Butenko, V.R.; Odegova, G. V.; Bulavchenko, O.A.; Nikolaeva, O.A.; Ozerova, A.M.; Lipatnikova, I.L.; Tayban, E.S.; Mukha, S.A.; et al. Dehydrogenation of Ammonia Borane Recrystallized by Different Techniques. *Renew. Energy* **2022**, *184*, 460–472, doi:10.1016/j.renene.2021.11.119.
3. Rueda, M.; Sanz-Moral, L.M.; Segovia, J.J.; Martín, Á. Enhancement of Hydrogen Release Kinetics from Ethane 1,2 Diamineborane (EDAB) by Micronization Using Supercritical Antisolvent (SAS) Precipitation. *Chem. Eng. J.* **2016**, *306*, 164–173, doi:10.1016/j.cej.2016.07.052.
4. Leardini, F.; Valero-Pedraza, M.J.; Perez-Mayoral, E.; Cantelli, R.; Bañares, M.A. Thermolytic Decomposition of Ethane 1,2-Diamineborane Investigated by Thermoanalytical Methods and *in Situ* Vibrational Spectroscopy. *J. Phys. Chem. C* **2014**, *118*, 17221–17230, doi:10.1021/jp501964r.
5. Li, Z.; He, T.; Liu, L.; Chen, W.; Zhang, M.; Wu, G.; Chen, P. Covalent Triazine Framework Supported Non-Noble Metal Nanoparticles with Superior Activity for Catalytic Hydrolysis of Ammonia Borane: From Mechanistic Study to Catalyst Design. *Chem. Sci.* **2016**, *8*, 781–788, doi:10.1039/C6SC02456D.
6. Li, H.; He, W.; Xu, L.; Pan, Y.; Xu, R.; Sun, Z.; Wei, S. Synergistic Interface between Metal Cu Nanoparticles and CoO for Highly Efficient Hydrogen Production from Ammonia Borane. *RSC Adv.* **2023**, *13*, 11569–11576, doi:10.1039/d3ra01265d.
7. Fu, F.; Wang, C.; Wang, Q.; Martinez-Villacorta, A.M.; Escobar, A.; Chong, H.; Wang, X.; Moya, S.; Salmon, L.; Fouquet, E.; et al. Highly Selective and Sharp Volcano-Type Synergistic Ni_2Pt@ZIF-8-Catalyzed Hydrogen Evolution from Ammonia Borane Hydrolysis. *J. Am. Chem. Soc.* **2018**, *140*, 10034–10042, doi:10.1021/JACS.8B06511/SUPPL_FILE/JA8B06511_SI_001.PDF.
8. Wang, Q.; Fu, F.; Escobar, A.; Moya, S.; Ruiz, J.; Astruc, D. "Click" Dendrimer-Stabilized Nanocatalysts for Efficient Hydrogen Release upon Ammonia-Borane Hydrolysis. *ChemCatChem* **2018**, *10*, 2673–2680, doi:10.1002/CCTC.201800407.
9. Zhao, Q.; Espuche, B.; Kang, N.; Moya, S.; Astruc, D. Cobalt Sandwich-Stabilized Rhodium Nanocatalysts for Ammonia Borane and Tetrahydroxydiboron Hydrolysis. *Inorg. Chem. Front.* **2022**, *9*, 4651–4660, doi:10.1039/d2qi01313d.
10. Paterson, R.; Alharbi, A.A.; Wills, C.; Dixon, C.; Šiller, L.; Chamberlain, T.W.; Griffiths, A.; Collins, S.M.; Wu, K.; Simmons, M.D.; et al. Heteroatom Modified Polymer Immobilized Ionic Liquid Stabilized Ruthenium Nanoparticles: Efficient Catalysts for the Hydrolytic Evolution of Hydrogen from Sodium Borohydride. *Mol. Catal.* **2022**, *528*, 112476, doi:10.1016/j.mcat.2022.112476.
11. Kang, N.; Wang, Q.; Djeda, R.; Wang, W.; Fu, F.; Moro, M.M.; Ramirez, M.D.L.A.; Moya, S.; Coy, E.; Salmon, L.; et al. Visible-Light Acceleration of H_2 Evolution from Aqueous Solutions of Inorganic Hydrides Catalyzed by Gold-Transition-Metal Nanoalloys. *ACS Appl. Mater. Interfaces* **2020**, *12*, 53816–53826, doi:10.1021/acsami.0c16247.
12. Meng, Y.; Sun, Q.; Zhang, T.; Zhang, J.; Dong, Z.; Ma, Y.; Wu, Z.; Wang, H.; Bao, X.; Sun, Q.; et al. Cobalt-Promoted Noble-Metal Catalysts for Efficient Hydrogen Generation from Ammonia Borane Hydrolysis. *J. Am. Chem. Soc.* **2023**, *145*, 5486–5495, doi:10.1021/JACS.3C00047/SUPPL_FILE/JA3C00047_SI_001.PDF.
13. Wang, C.; Tuninetti, J.; Wang, Z.; Zhang, C.; Ciganda, R.; Salmon, L.; Moya, S.; Ruiz, J.; Astruc, D. Hydrolysis of Ammonia-Borane over Ni/ZIF-8 Nanocatalyst: High Efficiency, Mechanism, and Controlled Hydrogen Release. *J. Am. Chem. Soc.* **2017**, *139*, 11610–11615, doi:10.1021/jacs.7b06859.
14. Song, J.; Gu, X.; Zhang, H. Electrons and Hydroxyl Radicals Synergistically Boost the Catalytic Hydrogen Evolution from Ammonia Borane over Single Nickel Phosphides under Visible Light Irradiation. *ChemistryOpen* **2020**, *9*, 366–373, doi:10.1002/OPEN.201900335.
15. Yang, L.; Liu, Z.; Qu, B.; Tao, Y.; Liu, Y. Highly Efficient Dehydrogenation of Ammonia Borane over Reduced Graphene Oxide-Supported Pd@NiP Nanoparticles at Room Temperature. *Int. J. Energy Res.* **2023**, *2023*, 1–13, doi:10.1155/2023/9889312.
16. Zhang, L.; Ye, J.; Tu, Y.; Wang, Q.; Pan, H.; Wu, L.; Zheng, X.; Zhu, J. Oxygen Modified CoP2 Supported Palladium Nanoparticles as Highly Efficient Catalyst for Hydrolysis of Ammonia Borane. *Nano Res.* **2022**, *15*, 3034–3041, doi:10.1007/S12274-021-3941-7/METRICS.
17. Chen, W.; Li, D.; Wang, Z.; Qian, G.; Sui, Z.; Duan, X.; Zhou, X.; Yeboah, I.; Chen, D. Reaction Mechanism and Kinetics for Hydrolytic Dehydrogenation of Ammonia Borane on a Pt/CNT Catalyst. *AIChE J.* **2017**, *63*, 60–65, doi:10.1002/aic.15389.
18. Wang, Q.; Fu, F.; Yang, S.; Martinez Moro, M.; Ramirez, M.D.L.A.; Moya, S.; Salmon, L.; Ruiz, J.; Astruc, D. Dramatic Synergy in CoPt Nanocatalysts Stabilized by "Click" Dendrimers for Evolution of Hydrogen from Hydrolysis of Ammonia Borane. *ACS Catal.* **2019**, *9*, 1110–1119, doi:10.1021/ACSCATAL.8B04498/SUPPL_FILE/CS8B04498_SI_001.PDF.
19. Fu, W.; Wang, Q.; Chen, W.; Qian, G.; Zhang, J.; Chen, D.; Yuan, W.-K.; Zhou, X.; Duan, X.; Yuan, W. Fabrication and Engineering of Ru Local Structures toward Enhanced Kinetics of Hydrogen Generation. *Authorea Prepr.* **2020**, *2*, doi:10.22541/AU.159908943.33972088.
20. Mohsenzadeh, A.; Bolton, K.; Richards, T. DFT Study of the Adsorption and Dissociation of Water on Ni(111), Ni(110) and Ni(100) Surfaces. *Surf. Sci.* **2014**, *627*, 1–10, doi:10.1016/j.susc.2014.04.006.
21. Cui, C.; Liu, Y.; Mehdi, S.; Wen, H.; Zhou, B.; Li, J.; Li, B. Enhancing Effect of Fe-Doping on the Activity of Nano Ni Catalyst towards Hydrogen Evolution from NH_3BH_3. *Appl. Catal. B Environ.* **2020**, *265*, 118612, doi:10.1016/J.APCATB.2020.118612.
22. Liu, R. Adsorption and Dissociation of H_2O on Au(1 1 1) Surface: A DFT Study. *Comput. Theor. Chem.* **2013**, *1019*, 141–145, doi:10.1016/J.COMPTC.2013.07.009.

23. Petersen, T.; Klüner, T. Water Adsorption on Ideal Anatase-TiO2(101) - An Embedded Cluster Model for Accurate Adsorption Energetics and Excited State Properties. *Zeitschrift fur Phys. Chemie* **2020**, *234*, 813–834, doi:10.1515/zpch-2019-1425.
24. Guan, S.; An, L.; Ashraf, S.; Zhang, L.; Liu, B.; Fan, Y.; Li, B. Oxygen Vacancy Excites Co3O4 Nanocrystals Embedded into Carbon Nitride for Accelerated Hydrogen Generation. *Appl. Catal. B Environ.* **2020**, *269*, 118775 (1-10), doi:10.1016/j.apcatb.2020.118775.
25. Zhao, W.; Bajdich, M.; Carey, S.; Vojvodic, A.; Nørskov, J.K.; Campbell, C.T. Water Dissociative Adsorption on NiO(111): Energetics and Structure of the Hydroxylated Surface. *ACS Catal.* **2016**, *6*, 7377–7384, doi:10.1021/ACSCATAL.6B01997/SUPPL_FILE/CS6B01997_SI_001.PDF.
26. Chen, W.; Zheng, W.; Cao, J.; Fu, W.; Qian, G.; Chen, D.; Zhou, X.; Duan, X. Atomic Insights into Robust Pt-PdO Interfacial Site-Boosted Hydrogen Generation. *ACS Catal.* **2020**, *10*, 11417–11429, doi:10.1021/acscatal.0c03214.
27. Chen, W.; Fu, W.; Qian, G.; Zhang, B.; Chen, D.; Duan, X.; Zhou, X. Synergistic Pt-WO_3 Dual Active Sites to Boost Hydrogen Production from Ammonia Borane. *iScience* **2020**, *23*, 100922, doi:10.1016/j.isci.2020.100922.
28. Wang, C.; Ren, Y.; Zhao, J.; Sun, S.; Du, X.; Wang, M.; Ma, G.; Yu, H.; Li, L.; Yu, X.; et al. Oxygen Vacancy-Attired Dual-Active-Sites Cu/$Cu_{0.76}Co_{2.24}O_4$ Drives Electron Transfer for Efficient Ammonia Borane Dehydrogenation. *Appl. Catal. B Environ.* **2022**, *314*, 121494 (1-12), doi:10.1016/j.apcatb.2022.121494.
